# Grain zinc concentrations differ among Brazilian wheat genotypes and respond to zinc and nitrogen supply

**DOI:** 10.1371/journal.pone.0199464

**Published:** 2018-07-10

**Authors:** João Augusto Lopes Pascoalino, Jacqueline A. Thompson, Gladys Wright, Francisco Assis Franco, Pedro Luiz Scheeren, Volnei Pauletti, Milton Ferreira Moraes, Philip John White

**Affiliations:** 1 Department of Soil Science, Federal University of Paraná, Curitiba, Paraná, Brazil; 2 Department of Ecological Sciences, The James Hutton Institute, Invergowrie, Dundee, United Kingdom; 3 Central Cooperative for Agricultural Research, Cascavel, Paraná, Brazil; 4 Nacional Center for Wheat Research of the Brazilian Agricultural Research Company, Passo Fundo, Rio Grande do Sul, Brazil; 5 Department of Tropical Agriculture, Federal University of Mato Grosso, Barra do Garças, Mato Grosso, Brazil; Institute of Genetics and Developmental Biology Chinese Academy of Sciences, CHINA

## Abstract

The combined application of nitrogen (N) and zinc (Zn) fertilizers is a promising agronomic strategy for the biofortification of wheat grain with Zn for human nutrition. A glasshouse experiment was carried out to assess the effects of supplying N on the uptake, translocation and accumulation of Zn in tissues of two wheat genotypes (Quartzo and BRS Parrudo) with contrasting potential for grain Zn biofortification. Winter wheat genotypes were grown to maturity in 5 cm diameter, 100 cm length tubes filled with a mixture of sand, grit and gravel (40:40:20 v/v/v) over a layer of 0.1 m^3^ of gravel, and supplied a full nutrient solution with low Zn (0.15 μM) or high Zn (2.25 μM) and low N (0.4 mM) or high N (4.0 mM) concentrations. High N supply increased biomass production, Zn concentration and Zn content of straw and grain in both Quartzo and BRS Parrudo. Grain Zn content more than doubled when the supplies of Zn and N were both increased from low to high in both genotypes. Quartzo had a greater grain yield than BRS Parrudo. BRS Parrudo had greater grain Zn concentration and Zn content than Quartzo. A greater N supply promoted better uptake, translocation to the shoot and accumulation of Zn within the grain. Quartzo and BRS Parrudo differed in their partitioning of biomass and Zn between tissues. It might be possible to combine the greater grain yield of Quartzo with the greater grain Zn accumulation of BRS Parrudo to deliver a greatly improved genotype for human food security.

## Introduction

Zinc (Zn) is an essential nutrient for humans. Zinc deficiency in humans can lead to impairment of the immune system combined with increased risk of infection, impaired physical growth and delayed learning ability [1, 2]. It is estimated that more than 20% of the world’s population has insufficient Zn in their diets [3, 4, 5, 6]. This is largely a consequence of consuming foods with low Zn content [5, 7].

The cereals, especially wheat, are the main source of calories in the majority of developing countries [8, 9, 10]. At present, three cereals, wheat, rice and maize, provide up to 60% of the daily energy intake of human populations [11], and bread wheat alone is the staple food for 35% of the world’s population [12]. Bread is eaten by about 95% of Brazilians, who, in 2013, consumed 145 g wheat products capita^-1^ d^-1^ [13]. However, wheat is inherently poor in mineral nutrients and has a low concentration and bioavailability of Zn in its grain [14, 15, 16]. This concentration can be < 10 mg kg^-1^ Zn in soils with low Zn phytoavailabilty [16, 17, 18]. Although the recommended dietary intake of Zn for humans is 12–15 mg d^-1^ [19] and the average Zn intake in Brazil is 19.3 mg capita^-1^ d^-1^ [20], between 3 and 20% of the Brazilian population is at risk from Zn deficiency [20, 21]. The production of cereal grains with greater Zn concentrations could increase Zn intakes and improve the nutritional status of the Brazilian population.

There are several approaches to increase Zn intakes by humans, including programs for supplementation and fortification [7, 22, 23]. In addition, crops can be biofortified with Zn by increasing the phytoavailability of Zn through agronomy and utilising genotypes that accumulate greater concentrations of Zn in their edible portions. Studies have shown that combining crop breeding and agronomic strategies is the most economic and sustainable approach to overcome human micronutrient deficiencies, including Zn deficiency [7, 9, 23, 24, 25, 26]. The application of fertilizer is fundamental to any agronomic approach to improve the yield and nutritional quality of food crops. The contribution of Zn fertilizer application to the accumulation of Zn in grain depends on both plant and soil factors, including nutrient and water availability and the nitrogen (N) nutritional status of the plant [16]. Wide genetic variation in grain Zn concentration has been observed in wheat [7, 27], indicating the existence of potential for genetic improvement.

The exploitation of positive interactions between N status and Zn accumulation appears to be a promising agronomic strategy to increase Zn concentration in wheat grain [16, 28, 29, 30, 31, 32]. It has been observed that the increase in grain Zn concentration produced by application of soil and foliar Zn fertilizers is enhanced by the addition of N fertilizer [33]. Studies varying N supply have revealed that optimizing N status of wheat can improve root Zn uptake, root-to-shoot translocation of Zn and Zn remobilization within the plant, all of which increase Zn accumulation in the grain [28, 34]. A better understanding of the influence of N on the uptake and distribution of Zn in wheat, as well as a detailed description of the behaviour of genotypes with distinct potentials for biofortification with Zn, will contribute to the effectiveness of agronomic and genetic biofortification strategies.

The objective of this study was to assess the effects of N application on the uptake, translocation and accumulation of Zn in tissues of two wheat genotypes with contrasting potential for grain Zn biofortification. Nutrient treatments consisted of four combinations of two rates of N supply, a rate that was sufficient for optimal plant growth and a greater N supply to investigate whether supra-optimal N supply could increase grain Zn concentration, and two rates of Zn supply, a rate that was sufficient for optimal plant growth and a greater Zn supply to investigate whether supra-optimal Zn supply could increase grain Zn concentration further.

## Materials and methods

### Plant material

**Fig 1:**
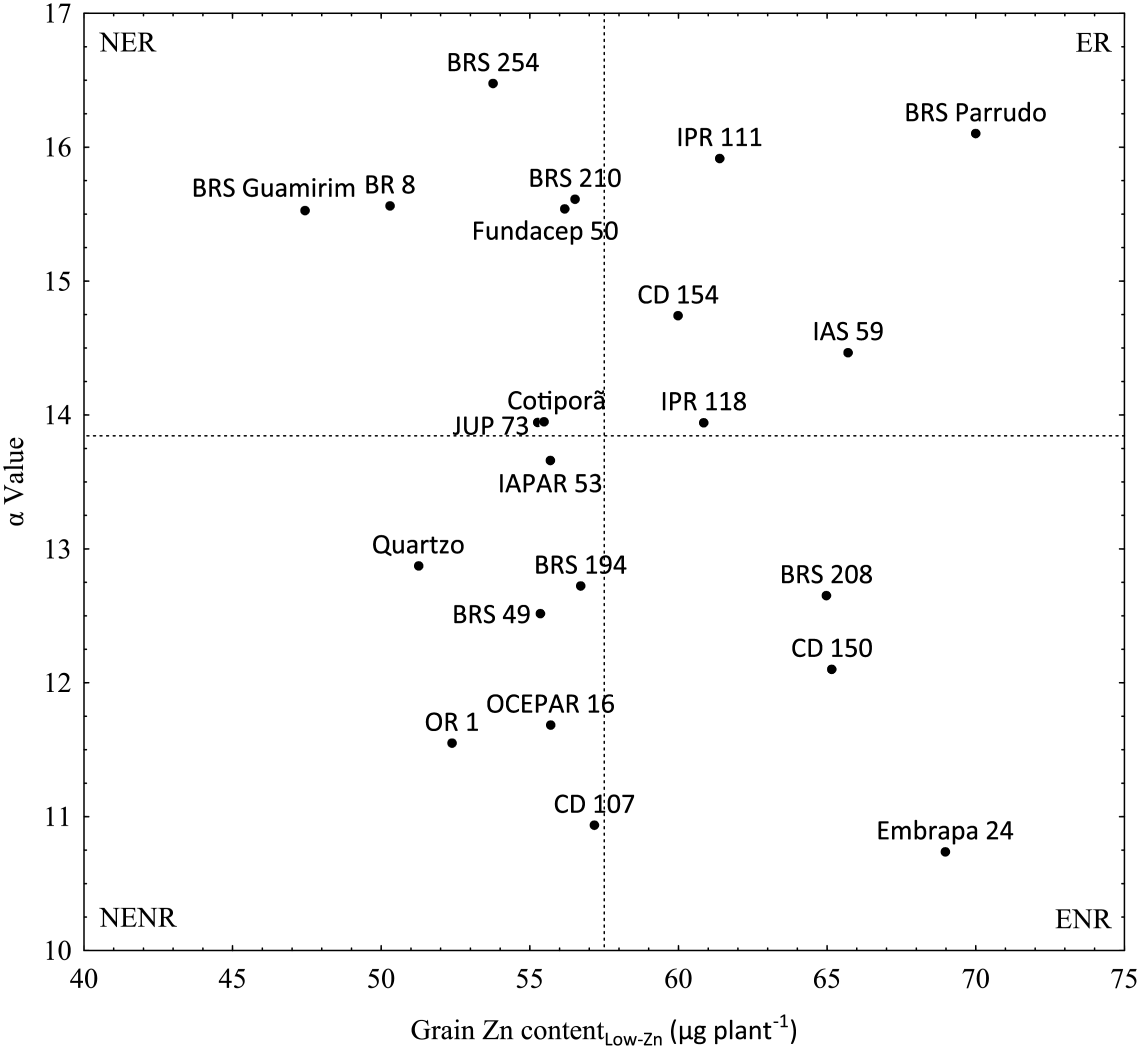
Classification of 22 winter wheat genotypes based on the relationship between grain Zn content (_Low-Zn_ = no Zn added in the soil) and the α value for grain Zn content calculated between applications of 0 and 5 mg Zn dm^-3^ [35]. The solid lines represent the mean value for the axis. NER = non-efficient and responsive genotypes, ER = efficient and responsive genotypes, NENR = non-efficient and non-responsive genotypes and ENR = efficient and non-responsive genotypes. Values represent the mean of four independent replicates.

Seeds of five winter wheat genotypes (Quartzo, CD 150, CD 154, BRS Guamirim and BRS Parrudo) were obtained from the seed stock of the Central Agricultural Research Cooperative (COODETEC, Brazil). Genotypes were chosen based on a preliminary study of the potential for biofortification of the grains of 22 winter wheat genotypes with Zn, which included varieties released between 1965 and 2012. Plants were grown to maturity with deficient (0 mg dm^-3^) and sufficient (5 mg dm^-3^) Zn in the soil. All genotypes were assayed for their grain Zn content. The metric of agronomic nutrient use efficiency (α), traditionally applied to identify more productive genotypes, was used to characterize the responses of grain Zn content to applications of Zn. It was calculated as α = [(Zn content in grain with high fertilization—Zn content in grain with low fertilization) / difference between the fertilizer application rates] [35]. Genotypes were separated into four groups: efficient and responsive–ER, efficient and not responsive–ENR, not efficient and responsive–NER, and not efficient and not responsive–NENR (Fig 1). Of the five genotypes grown in this study, the nutritional status of only Quartzo and BRS Parrudo was analysed. Quartzo was classified as a NENR genotype, while BRS Parrudo was classified as an ER genotype.

### Growing conditions

Seeds were screened (passed through a mesh of 3 mm, retained in a mesh of 2.5 mm) to standardize their size. The seeds were soaked in water for 3 to 5 hours and then surface sterilized for 15 minutes in 2% (w/v) calcium hypochlorite. The sterilized seeds were rinsed several times in distilled water and placed between sheets of moist germination paper (Anchor Paper Company, St Paul, MN, USA) in Petri dishes (20 cm x 25 cm). The dishes were then covered with aluminum foil and incubated at 15 °C for 5 days until the roots of germinated seeds reached 5 to 10 mm in length. Five days after sowing, on May 21, 2016, germinated seedlings with similar root and leaf development were selected and transfered to tubes containing a substrate of sand, grit and gravel.

Plants were cultivated in plastic tubes (5 cm in diameter and 100 cm in length) lined with black plastic sheet and filled with a substrate of sand, grit and gravel (40:40:20 v/v/v) over a layer of 0.1 m^3^ of gravel to facilitate drainage of water. The bottom of each tube was covered with nylon net with a pore size of 5 mm to prevent loss of substrate. Homogeneity of the substrate was obtained using a drum compost mixer (720 rpm for 10 min). The content of each tube was carefully moistened with water to allow the substrate to settle before transplanting pairs of germinated seedlings to each tube. After establishment of the seedlings, one seedling was removed from each tube.

Plants were grown in a Cambridge type compartment in a glasshouse at the James Hutton Institute (Invergowrie, Scotland, UK, latitude 56°27'22.7" N, longitude 03°04'09.9" W). Daytime and nighttime temperatures were maintained at 24 °C and 18 °C, respectively. Daily irradiance (> 200 W m^-2^ for 16 h) was obtained by artificial illumination (MASTER SON-T PIA Green Power lamps, Philips, Guildford, UK). The experimental design was completely randomized with eight blocks. Each block contained one replicate of each genotype for all four nutrient treatments. Thus, there were eight replicates of each genotype for each nutrient treatment. The tubes were arranged in 40 rows and four columns, and five rows constituted a block.

### Nutrient treatments and fertirrigation

Nutrient treatments consisted of combinations of solutions containing ZnSO_4_ and NH_4_NO_3_ with concentrations of 0.15 μM (sufficient = low Zn) or 2.25 μM (supra-sufficient = high Zn) and 0.40 mM (sufficient = low N) or 4.00 mM (supra-sufficient = high N), respectively. The treatments were applied weekly, using a Multipette^®^ M4 (Combitip system). Other nutrients were applied using an automatic irrigation system (HortiMaX Growing Solutions Aqua 500 Irrigation Computer, HortiMaX B.V., Netherlands) connected to a nutrient reservoir through a Dosatron DI 16 dosing pump (Dosatron International, Bordeaux, France), and delivered to each plant via drippers inserted in the substrate of each tube, at an administration rate of 9.0 mL min^-1^. The reservoir contained a nutrient solution with final concentrations of 0.50 mM K_2_HPO_4_, 0.51 mM K_2_SO_4_, 0.75 mM MgSO_4_, 10.00 μM H_3_BO_3_, 1.00 μM MnSO_4_, 0.25 μM CuSO_4_, 0.25 μM MoO_3_, 0.06 μM Na_2_SeO_3_, 0.06 μM CoSO_4_, 0.06 μM NiSO_4_ and 10.00 μM FeNaEDTA. Fertirrigation was applied daily at a rate of 45 mL tube^-1^ during the first four weeks after transplanting seedlings to the tubes, and 72 mL tube^-1^ thereafter until the end of the experiment. Separately, 16.5 mL tube^-1^ of 1.25 mM CaCl_2_ was applied weekly during the first four weeks and 27.0 mL tube^-1^ of the same solution subsequently. The total quantity of liquid applied to all tubes was equal and all the nutrient solutions were prepared with distilled water, with pH between 5.7 and 5.9. Nutrient treatments and fertirrigation were performed until maturity (Z92; [36]), when the supply of nutrients and water were gradually decreased to nothing at the time of harvesting.

### Harvesting and measurements

The plants were harvested on September 10, 2016. Aboveground plant parts were removed and separated into straw (stem, leaf and chaff) and grain. Roots were removed by sliding out the polythene sheet lining from each tube. Roots were washed with water to separate them from the substrate. Each plant part (roots, straw and grain) was weighed separately to obtain its fresh weight (FW), and then each part was dried at 70 °C in an oven for 3 days to determine its dry weight (DW).

### Mineral analysis

Dry plant material was ground to a fine powder and acid digested in a closed microwave system (MarsExpress, CEM., Buckingham, UK) as described by White et al. [37]. Following digestion, the samples were diluted to a final volume of 50 mL with Milli-Q water. Zinc concentrations were determined by inductively coupled plasma-mass spectrometry (ICP-MS ELAN DRCe, PerkinElmer, Waltham, MA, USA) and the measurements were verified using a tomato leaf standard (Reference 1573a) from National Institute of Standards and Technology (NIST, Gaithersburg, MD, USA).

### Statistical analysis

To calculate the Zn content of a plant part (root, straw, grain), its Zn concentration was multiplied by the dry weight (DW) of that part. Data were submitted to one-way or two-way analysis of variance (ANOVA) to evaluate the significance of the effects of the treatments and their interactions on the characteristics analyzed. When the effects were significant according to the ANOVA, significant differences between the mean values were determined by the Tukey test at 95% confidence (*P* < 0.05).

## Results

Biomass and Zn content of root, straw and grain of two winter wheat genotypes, Quartzo and BRS Parrudo, with contrasting grain Zn biofortification characteristics (Fig 1) were studied in detail. The genotypes were grown under combinations of treatments of Zn (Low-Zn: 0.15 μM or High-Zn: 2.25 μM) and N (Low-N: 0.4 mM or High-N: 4.0 mM). The ANOVA revealed significant effects of genotype, supply of Zn and N, and their respective interactions on the root dry weight, straw dry weight, grain yield and total plant dry weight (designated Root DW, Straw DW, Grain yield and Total DW), Zn concentration (designated Root [Zn], Straw [Zn], Grain [Zn] and Total [Zn]), Zn content (designated Root ZnC, Straw ZnC, Grain ZnC and Total ZnC), grain and Zn harvest index (designated GHI and GZnHI, respectively), and the ratio between straw and root dry weight (designated Straw:root ratio) (Table 1).

**Table 1:** Percentage contribution from analysis of variance (ANOVA) for the effects of genotype (G), Zn supply (Zn), N supply (N) and their respective interactions on the dry weight (designated Root DW, Straw DW, Grain yield and Total DW), Zn concentration (designated Root [Zn], Straw [Zn], Grain [Zn] and Total [Zn]), Zn content (designated Root ZnC, Straw ZnC, Grain ZnC and Total ZnC), Grain and Grain Zn harvest index (designated GHI and GZnHI) and Straw:root ratio of winter wheat grown under glasshouse conditions.

Source of variation	df	Root DW	Root [Zn]	Root ZnC	Straw DW	Straw [Zn]	Straw ZnC	Grain yield	Grain [Zn]	Grain ZnC	Total DW	Total [Zn]	Total ZnC	Straw:root ratio	Harvest Index	
															GHI	GZnHI
		--------------------------------------------------------------------------------%------------------------------------------------------------------------------------														
Replication	7	--	--	--	--	--	--	--	--	--	--	--	--	--	--	--
G	1	81.8**	80.0**	46.0**	51,1**	20.5**	30.9**	54.5**	68.0**	33.9**	27.9**	20.2**	28.9**	60.9**	82,5**	52.0**
Zn	1	3.1**	15.4**	43.3**	2,1ns	42.3**	25.9**	2.4ns	15.2**	17.2**	5.3**	40.1**	24.4**	0.3ns	0,1ns	1.0ns
N	1	1.5**	0.5ns	0.2ns	43,1**	33.2**	39.2**	44.1**	26.0**	45.2**	48.5**	35.1**	43.3**	37.9**	15,5**	40.1**
G x Zn	1	0,1ns	0.9ns	0.4ns	0,1ns	0.2ns	0.4ns	0,0ns	0.0ns	0.0ns	0.1ns	0.1ns	0.1ns	0.0ns	0,0ns	3.2ns
G x N	1	0,0ns	0.0ns	0.2ns	3,5ns	0.7ns	2.4*	2.9*	0.4ns	3.2**	5.0**	1.2ns	3.0**	0.0ns	1,1ns	0.1ns
Zn x N	1	0,7ns	2.9**	10.0**	0,2ns	2.1*	0.7ns	0.1ns	0.2ns	0.0ns	0.1ns	2.7**	0.2ns	0.6ns	0,2ns	2.9ns
G x Zn x N	1	0,1ns	0.3ns	0.5ns	0,0ns	0.0ns	0.0ns	0.1ns	0.6ns	0.2ns	0.1ns	0.5ns	0.1ns	0.3ns	0,0ns	1.1ns

df: degree of freedom and ns,

* and ** are non-significant, significant at (0.01 < *P* ≤ 0.05) and (*P* ≤ 0.01), respectively.

### Plant dry weight

All the characteristics evaluated were affected significantly by genotype (*P* < 0.01), confirming the contrasting phenotypes of the genetic materials chosen for this study (Table 1). Root DW (*P* < 0.01) and Total DW (*P* < 0.01) were affected by both Zn and N supply, while Straw DW (*P* < 0.01), Grain yield (*P* < 0.01), Straw:root ratio (*P* < 0.01) and GHI (*P* < 0.01) were only affected by N supply (Table 1). Interactions between genotype and N supply were found for Grain yield (0.01 ≤ *P* < 0.05) and Total DW (*P* < 0.01) (Table 1). Although genotype and nutrient supply had strong effects on dry weight of tissues, the variation attributed to each differed (Table 1). The variation attributed to genotype (ranging from 27.9%– 82.5%) and N supply (1.5%– 48.5%) were much greater than the variation attributed to Zn supply (0.1%– 5.3%) (Table 1).

Plants supplied High-Zn with Low-N had greatest Root DW (Table 2). Plants supplied Low-Zn with High-N or High-Zn with High-N had greatest Straw DW, Grain yield and Total DW (Table 2). They also had the greatest Straw:root ratio and GHI (Table 2). Quartzo produced greater Grain yield, and had a larger Straw:root ratio and GHI than BRS Parrudo, whereas BRS Parrudo had greater Root DW and Straw DW than Quartzo (Table 2).

**Table 2:** Effects of Zn (Low: 0.15 μM or High: 2.25 μM) and N (Low: 0.4 mM or High: 4.0 mM) supply on dry weight production (designated Root DW, Straw DW, Grain yield and Total DW), straw:root ratio and grain harvest index (GHI) of two contrasting winter wheat genotypes (Quartzo and BRS Parrudo) grown under glasshouse conditions.

Genotype	Zn supply	N supply	Root DW	Straw DW	Grain yield	Total DW	Straw:Root ratio	GHI
			g plant^-1^	g plant^-1^	g plant^-1^	g plant^-1^	--	%
Quartzo	Low	Low	1.16b	5.05a	4.43b	10.63b	4.37b	41.72b
		High	1.13b	5.51a	5.14a	11.78a	4.88ab	43.69ab
	High	Low	1.25a	5.20a	4.60b	11.04b	4.17b	41.63b
		High	1.13b	5.58a	5.32a	12.02a	4.97a	44.23a
Mean			1.17B	5.33B	4.87A	11.4B	4.60A	42.82A
BRS Parrudo	Low	Low	1.59b	5.51b	3.63b	10.73b	3.46b	33.82b
		High	1.53c	6.29a	4.67a	12.49a	4.11a	37.38a
	High	Low	1.68a	5.68b	3.75b	11.12b	3.38b	33.76b
		High	1.57b	6.41a	4.92a	12.90a	4.07a	38.08a
Mean			1.60A	5.97A	4.24B	11.8A	3.76B	35.76B

Values are means (n = 8). Different upper-case letters are significantly different between genotypes and different lower-case letters are significantly different between the combinations of Zn and N supplementation according to One-way and Two-way ANOVA, respectively, followed by the Tukey’s test (*P* < 0.05).

### Concentration of Zn in plant tissues

The Straw [Zn] (*P* < 0.01), Grain [Zn] (*P* < 0.01) and Total [Zn] (*P* < 0.01) were affected by both Zn and N supply, while Root [Zn] (*P* < 0.01) was affected by Zn supply, but not by N supply (Table 1). Interactions between Zn and N supply were found for Root [Zn] (*P* < 0.01), Straw [Zn] (0.01 ≤ *P* < 0.05) and Total [Zn] (*P* < 0.01) (Table 1). The variation in the concentration of Zn in plant tissues attributed to genotype (20.0%– 80.0%), Zn supply (15.2%– 47.9%) and N supply (26.0%– 33.2%) were all high, and the variation attributed to the interaction between Zn and N supply contributed little to the results (0.2%– 2.9%) (Table 1).

Plants supplied High-Zn with Low-N or High-Zn with High-N had greater Root [Zn], and plants supplied High-Zn with High-N had greatest Straw [Zn], Grain [Zn] and Total [Zn] (Table 3). Quartzo had greater Root [Zn] than BRS Parrudo, whereas BRS Parrudo had greater Straw [Zn], Grain [Zn] and Total [Zn] than Quartzo. BRS Parrudo had on average 15.6%, 84.7% and 33.9% greater Straw [Zn], Grain [Zn] and Total [Zn] than the Quartzo, respectively (Table 3). The greater Grain [Zn] and greater increase in Grain [Zn] in response to increasing Zn supply at an optimal N supply (Table 3) is consistent with the results of the preliminary field trial (Fig 1).

**Table 3:** Effects of Zn (Low: 0.15 μM or High: 2.25 μM) and N (Low: 0.4 mM or High: 4.0 mM) supply on the Zn concentration (designated Root [Zn], Straw [Zn], Grain [Zn] and Total [Zn]), Zn content (designated Root ZnC, Straw ZnC, Grain ZnC and Total ZnC) and grain Zn harvest index (GZnHI) of two contrasting winter wheat genotypes (Quartzo and BRS Parrudo) grown under glasshouse conditions.

Genotype	Zn supply	N supply	Root [Zn]	Straw [Zn]	Grain [Zn]	Total [Zn]	Root ZnC	Straw ZnC	Grain ZnC	Total ZnC	GZnHI
			μg g^-1^ DW	μg g^-1^ DW	μg g^-1^ DW	μg g^-1^ DW	μg	μg	μg	μg	%
Quartzo	Low	Low	31.75c	31.60c	30.95c	31.30c	36.74c	160.76c	137.31c	334.81c	40.85b
		High	37.45b	41.86b	43.07b	41.42b	42.35bc	231.09ab	219.57b	493.01b	44.26ab
	High	Low	46.14a	43.63b	44.40b	44.33b	57.58a	227.04b	204.59b	489.21b	41.63b
		High	43.04a	49.96a	58.81a	53.09a	48.52b	279.28a	310.41a	638.21a	48.64a
Mean			39.60A	41.76B	44.31B	42.54B	46.30A	224.54B	217.97B	488.81B	43.85B
BRS Parrudo	Low	Low	17.72b	35.68c	61.83c	41.54c	28.26c	196.88c	224.98d	450.13d	49.90b
		High	21.47ab	49.39b	85.46ab	59.49b	32.90bc	310.72b	399.65b	743.28b	54.62a
	High	Low	26.14a	50.05b	83.35b	57.68b	44.02a	284.10b	313.09c	641.21c	48.77b
		High	25.06a	58.00a	96.78a	68.64a	39.47ab	371.52a	474.91a	885.90a	54.47a
Mean			22.60B	48.28A	81.86A	56.94A	36.17B	290.81A	353.16A	680.13A	52.94A

Values are means (n = 8). Different upper-case letters are significantly different between genotypes and different lower-case letters are significantly different between the combinations of Zn and N supplementation according to One-way and Two-way ANOVA, respectively, followed by the Tukey’s test (*P* < 0.05).

### Zn content of plant tissues

Straw ZnC (*P* < 0.01), Grain ZnC (*P* < 0.01) and Total ZnC (*P* < 0.01) were affected by Zn and N supply, while Root ZnC (*P* < 0.01) and GZnHI (*P* < 0.01) were affected only by Zn supply and N supply, respectively (Table 1). Interactions between genotype and N supply were found for Straw ZnC (0.01≤ *P* <0.05), Grain ZnC (*P* < 0.01) and Total ZnC (*P* < 0.01), while interaction between Zn and N supply was only observed for Root ZnC (*P* < 0.01) (Table 1). The variation in tissue Zn content attributed to genotype (28.9%– 52.0%), Zn supply (1.0%– 43.3%) and N supply (0.2%– 45.2%) were high, and the variation attributed to interactions between genotype and N supply (0.1%– 3.2%) and Zn and N supply (0.0–10%) was small (Table 1).

Supply of High-Zn with Low-N resulted in the largest Root ZnC, while supply of High-Zn with High-N gave the largest Straw ZnC, Grain ZnC and Total ZnC. Supply of Low-Zn with High-N or High-Zn with High-N promoted large GZnHI (Table 3). There was no difference between the supply of Low-Zn with High-N or High-Zn with Low-N in the concentration or content of Zn in the straw or grains (Table 3), except for the Grain ZnC of the BRS Parrudo genotype, for which the effect of supplying Low-Zn with High-N was greater than supplying High-Zn with Low-N. Quartzo had grater Root ZnC than BRS Parrudo, whereas BRS Parrudo had greater Straw ZnC, Grain ZnC, Total ZnC and GZnHI. BRS Parrudo had 29.5%, 62.0% and 39.1% greater Straw ZnC, Grain ZnC and Total ZnC than Quartzo, respectively (Table 3). The GZnHI of BRS Parrudo was 20.7% greater than that of Quartzo (Table 3).

## Discussion

In the experiment reported here, the N supply affected the production of biomass more than the Zn supply (Tables 1 and 2). Compared to the Low-Zn with Low N treatment, supplying plants High-Zn with Low-N increased Root DW up to 8% for Quartzo and 6% for BRS Parrudo (Table 2). Supplying plants High-N, irrespective of Zn supply, increased Straw DW and Grain yield by up to 11% and 20% for Quartzo and 16% and 36% for BRS Parrudo, respectively, but reduced the Root DW by up to 3% for Quartzo and 4% for BRS Parrudo (Table 2). These findings are consistent with the results of [28] for wheat, and data for other plant species, which indicate that shoot biomass increases and root biomass decreases when N supply is increased, leading to greater shoot/root biomass ratios [38]. Kutman et al. [34, 29] and Xue et al. [30] found that increasing N supply produced large increases in straw dry matter and grain yield. Furthermore Kutman et al. [34] reported that combining a low Zn supply with high N supply resulted in a 73% increase in grain yield, but combining a high Zn supply with a high N supply caused an increase of more than 350% in the grain yield. In the study reported here, the effects of Zn and N supplies on biomass production were less than those reported by Kutman et al. [34] or Xue et al. [30]. This is probably because the concentrations of Zn and N used here corresponded to sufficient (Low-Zn, Low-N) and supra-sufficient (High-Zn, High-N) supply for the growth of wheat plants. In previous studies, the lowest Zn and N supplies were insufficient for optimum plant growth, which would result in a greater responses to Zn and N applications.

Although BRS Parrudo had a greater response in Grain yield to N supply (increase of up to 36%), its productivity was still less than that of Quartzo (Table 2). This indicates that Quartzo has better biomass partitioning than BRS Parrudo, allocating 43% of the total biomass to grain production, compared to only 36% in BRS Parrudo (Table 2). This corroborates previous findings that genotypes with high potential for biofortification, like BRS Parrudo, often have less grain yield but greater concentration and content of mineral nutrients [39, 40].

Root [Zn] was influenced strongly by Zn supply, while Straw [Zn] and Grain [Zn] were influenced by the supply of both Zn and N (Tables 1 and 3). The increases in Root [Zn], Straw [Zn] and Grain [Zn] observed when Zn supply was increased were up to 45%, 58% and 90% for Quartzo and 47%, 57% and 63% for BRS Parrudo, respectively (Table 3). High Zn with High N supply resulted in the greatest concentration of Zn in all plant tissues (Table 3), which suggests that providing a High-N supply to wheat plants improves both their uptake of Zn and their transport of Zn from the roots to the shoot. Erenoglu et al. [28], Kutman et al. [34, 29] and Barunawati et al. [31] have also reported that increasing N supply to wheat significantly increases Zn uptake by roots and the transport of Zn to the shoot. It is possible that increasing plant N supply increased the abundance of proteins and metabolites associated with the uptake of Zn by roots and the transport of Zn within plants [27, 41, 42, 43]. The main N-compounds associated with the transport of Zn in the xylem are Zn-asparagine, Zn-histidine and Zn-nicotianamine [7, 27].

**Fig 2:**
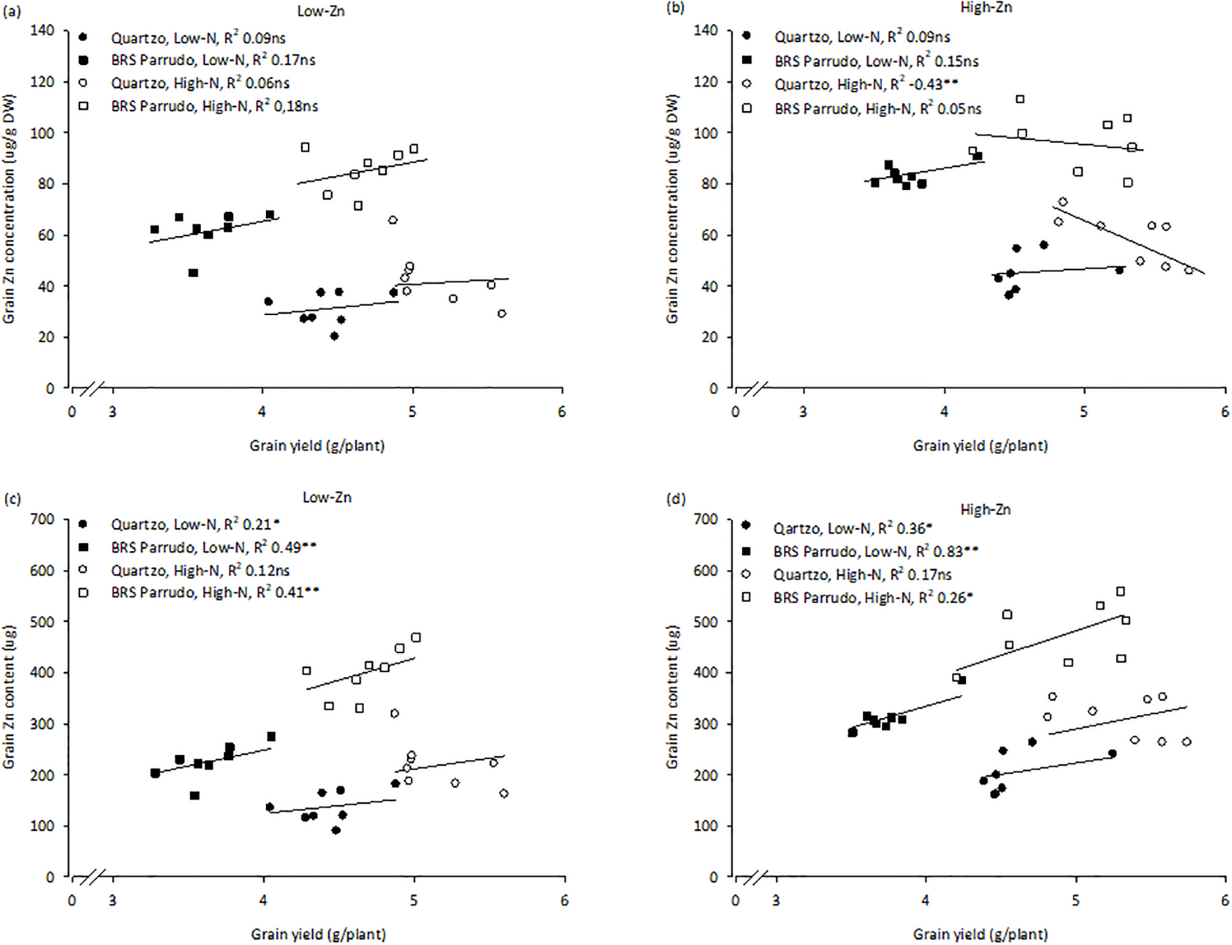
Relationships between grain yield and grain Zn concentration (a, c) and grain yield and grain Zn content (b, d) of two winter wheat genotypes (Quartzo and BRS Parrudo) grown with a low Zn (a, c; 0.15 μM) or high Zn (b, d; 2.25 μM) supply and either a low N (0.4 mM) or high N (4.0 mM) supply. ns, * and ** indicate non-significant, significant at (0.01 < *P* ≤ 0.05) and (*P* ≤ 0.01), respectively.

The responses in tissue Zn concentrations of Quartzo and BRS Parrudo to Zn and N supply were similar (Table 3). However, BRS Parrudo had greater Straw [Zn] and Grain [Zn] than Quartzo (Table 3), suggesting that BRS Parrudo is better able to transport Zn from the roots to the shoot (straw plus grain). Across all treatments, BRS Parrudo directed 95% of the total Zn in the plant (680 μg to the shoot, and, of this, 5% was mobilized to the grain, while Quartzo directed 91% of the total Zn in the plant (489 μg) to the shoot, and, of this, only 45% of the Zn was translocated to the grain (Table 3). Although differences were found for Grain yield and Grain [Zn] between the treatments and genotypes (Tables 2 and 3), no significant correlation was found between these variables, except for Quartzo in High-Zn with High-N treatment (r^2^ = 0.43; *P* <0.05) in which grain Zn concentration decreased with increasing grain yield (Fig 2a and 2b). This suggests that there is no general rule for the occurrence of nutrient dilution in genotypes with high yield as discussed by White et al. [37], who also observed that the concentration of minerals in tubers of high-yielding potato genotypes can be increased by the application of mineral fertilizers without compromising yield. Thus, it is possible to achieve more nutritious produce without compromising yield.

Increasing the supply of both Zn and N increased the content of Zn in plant tissues. Increases of Root ZnC, Straw ZnC and Grain ZnC were up to 57%, 74% and 126% for Quartzo and 56%, 89% and 130% for BRS Parrudo, respectively. The treatments that provided the greatest increases were High-Zn with Low-N for Root ZnC and High-Zn with High-N for Straw ZnC and Grain ZnC in both genotypes (Table 3). Additionally, the Grain ZnC more than doubled when the supplies of Zn and N were both increased from low to high in both genotypes (Table 3). This agrees with the results of Kutman et al. [33], who observed that increasing N supply increased the Zn content of grains when the supply of Zn was not limited. The increases in Zn content in the roots, straw and grains (Table 3) were much greater than the increases in dry matter of these tissues (Table 2) when N supply was increased. Apparently the greater Zn content in the plant was due to greater Zn uptake and translocation of Zn from the roots to the shoot, possibly as a consequence of greater nutritional demand. Thus, the uptake of Zn per unit of biomass increased as the shoot/root biomass quotient increased (Tables 2 and 3) and Zn uptake and translocation of Zn to the shoot was maintained or increased when the N supply was increased. This is corroborated by the fact that Grain yield was significantly and positively correlated with Grain ZnC in both genotypes in all treatments, except for Quartzo with a High N supply (Fig 2c and 2d). Moreover, the correlations between Grain yield and Grain ZnC were stronger at Low-N supply than at High-N supply, although High-N supply promoted greater accumulation of Zn in the grains, providing evidence of the positive effect of N nutrition on Zn movement to the grain. This effect might be explained because the remobilization of Zn from leaves to grain occurs mainly through the phloem in the form of N compounds [27]. The transport of Zn to cereal grain can be enhanced by greater production of nicotianamine, which chelates Zn in the phloem [44, 45, 32].

Regarding the partitioning of Zn in the plant, BRS Parrudo accumulated 97% of its total Zn (680 μg plant^-1^) in the shoot, of which 54% was in the grains (Table 3). Quartzo, by contrast, accumulated 90% of its total Zn (489 μg plant^-1^) in the shoot, of which only 45% was present in the grains (Table 3). The greater Grain [Zn] and Grain ZnC of BRS Parrudo than Quartzo (Tables 2 and 3), might be utilized for breeding genotypes for Zn biofortification.

## Conclusions

A greater N supply promoted better Zn uptake, translocation of Zn to the shoot and accumulation of Zn in the grain of wheat plants. Thus, management of N nutritional status is a promising route for the biofortification of wheat grain with Zn. Although this study was conducted under controlled conditions, field experiments have been conducted demonstrating the same synergistic effect of N on Zn uptake and accumulation in cereals grains, supporting the hypothesis that effective Zn biofortification of wheat grain is provided by the judicious application of both N and Zn fertilizer. The genotypes analyzed in the experiment reported here showed large contrasts in their partitioning of biomass and Zn between plant parts. Since there was no negative relationship between grain yield and grain Zn concentration or content, it might be possible combine the greater grain yield of the Quartzo genotype with the better Zn partitioning in the BRS Parrudo genotype to deliver a greatly improved genotype. This would be a significant advance for the biofortification of wheat for human nutrition.

## Supporting information

S1 Table Grain yield, Zn content and Zn concentration, and α values [α = (Zn content in grain with high fertilization—Zn content in grain with low fertilization) / difference between the fertilizer application rates] for 4 paired replicates of 22 winter wheat genotypes cultivated in soils to which 0 or 5 mg Zn dm^-3^ had been applied. (XLSX)

S2 Table Effects of zinc (Zn) and nitrogen (N) supply on the dry weight (DW), Zn concentration ([Zn]) and Zn content of roots, straw and grain of wheat genotypes Quartzo and BRS Parrudo grown to maturity under glasshouse conditions. (XLSX)
